# Scald resistance in hybrid rye (*Secale cereale*): genomic prediction and GWAS

**DOI:** 10.3389/fpls.2024.1306591

**Published:** 2024-01-18

**Authors:** Mette Dam Madsen, Peter Skov Kristensen, Khalid Mahmood, Tine Thach, Marius Mohlfeld, Jihad Orabi, Pernille Sarup, Ahmed Jahoor, Mogens Støvring Hovmøller, Julian Rodriguez-Algaba, Just Jensen

**Affiliations:** ^1^ Centre for Quantitative Genetic and Genomics, Faculty of Science and Technology, Arhus University, Aarhus, Denmark; ^2^ Research and Development Department, Nordic Seed A/S, Dyngby, Denmark; ^3^ Department of Agroecology, Faculty of Technical Sciences, Aarhus University, Slagelse, Denmark; ^4^ Department of Breeding, Nordic Seed A/S, Nienstädt, Germany

**Keywords:** rye, Secale cereale, scald, Rhynchosporium secalis, hybrid, GWAS, genomic prediction

## Abstract

Rye (*Secale cereale L*.) is an important cereal crop used for food, beverages, and feed, especially in North-Eastern Europe. While rye is generally more tolerant to biotic and abiotic stresses than other cereals, it still can be infected by several diseases, including scald caused by *Rhynchosporium secalis*. The aims of this study were to investigate the genetic architecture of scald resistance, to identify genetic markers associated with scald resistance, which could be used in breeding of hybrid rye and to develop a model for genomic prediction for scald resistance. Four datasets with records of scald resistance on a population of 251 hybrid winter rye lines grown in 2 years and at 3 locations were used for this study. Four genomic models were used to obtain variance components and heritabilities of scald resistance. All genomic models included additive genetic effects of the parental components of the hybrids and three of the models included additive-by-additive epistasis and/or dominance effects. All models showed moderate to high broad sense heritabilities in the range of 0.31 (SE 0.05) to 0.76 (0.02). The model without non-additive genetic effects and the model with dominance effects had moderate narrow sense heritabilities ranging from 0.24 (0.06) to 0.55 (0.08). None of the models detected significant non-additive genomic variances, likely due to a limited data size. A genome wide association study was conducted to identify markers associated with scald resistance in hybrid winter rye. In three datasets, the study identified a total of twelve markers as being significantly associated with scald resistance. Only one marker was associated with a major quantitative trait locus (QTL) influencing scald resistance. This marker explained 11-12% of the phenotypic variance in two locations. Evidence of genotype-by-environment interactions was found for scald resistance between one location and the other two locations, which suggested that scald resistance was influenced by different QTLs in different environments. Based on the results of the genomic prediction models and GWAS, scald resistance seems to be a quantitative trait controlled by many minor QTL and one major QTL, and to be influenced by genotype-by-environment interactions.

## 1 Introduction

Rye (*Secale cereale* L.) is a commercially important cereal crop used for bread and other baked goods, alcohol production, and for livestock feed. In 2021, 68% of the world’s rye was produced in the following countries: Germany (25%), Poland (19%), Russia (13%), Belarus (6%) and Denmark (5%) (FAOSTAT, 2023). This distribution is primarily due to the remarkable winter hardiness of rye, as well as its ability to tolerate multiple biotic and abiotic stresses and to thrive in low nutrient sandy soils compared to other cereals (Bushuk, 2001; Miedaner and Laidig, 2019). In Europe, approximately 40% of the rye produced is used for human consumption (European Commission, 2023), making it an integral part of the food culture of these regions.

While rye is considered a generally healthy crop, it can still be affected by several diseases. One common disease is scald. The impact of scald infection on yield in rye has to our knowledge not been reported in the literature, but yield reductions of up to 48% have been observed in barley (Khan, 1986; Kavak, 2004; McLean and Hollaway, 2018). In rye and triticale, scald is caused by the necrotrophic pathogen *Rhynchosporium secalis* sensu stricto (Oudem.) J.J. Davis (Caldwell, 1937; Zaffarano et al., 2008). The majority of scientific literature and studies consider *R. secalis* (sensu lato) as a monophyletic group with broad host range, mainly focusing on barley (*Hordeum vulgare*) as host and less on rye (Zhan et al., 2008). In recent years, the fungus causing scald on barley has been considered as a separate species from *R. secalis* and is now named *R. graminicola* Heinsen (before that *R. commune*) (Zaffarano et al., 2011; Crous et al., 2021). Typical leaf symptoms of scald on rye are water-soaked lesions of lenticular shape with a white-grey center and dark-brown margin (Caldwell, 1937). Conidia grow within the lesions and have a characteristic shape, curved at the tip (beak-like) and between 10.8-19.8µm in length. The pathogen is soil-borne, capable of surviving in infected stubble, and can infect susceptible host varieties in autumn, spring, and early summer. The sporulation of *R. secalis* requires high relative humidity (around 95%) and occurs at optimum temperatures between 15-20°C. The fungus is well adapted to temperate climate and can sporulate at temperatures down to 5°C (Caldwell, 1937).

Scald is often managed by fungicides, which require timely application and sometimes repeated treatments for effective control. However, this increases production costs and the potential risks of undesired residues in products, and thereby negative environmental impacts. Many consumers, therefore, oppose the use of fungicides, and the European Green Deal has proposed to reduce the use of chemical pesticides by 50% by 2030 to deliver on the Farm to Fork strategy (European Commission, 2022). As the use of fungicides is reduced, producers of rye and other crops will have to rely more on genetic disease resistance. This can be done through selective breeding of varieties with high resistance to fungal infections such as scald.

Access to genomic information has accelerated the breeding of more resistant crops through methods such as marker assisted selection (MAS) and genomic selection (GS). The optimal (combination of) method(s) depend on the genetic architecture of the disease resistance trait in question (Poland and Rutkoski, 2016). To our knowledge no previous investigations of the genetic architecture of scald resistance in rye have been published. However, generally the genetic architecture of disease resistance falls somewhere on the spectrum from being qualitative resistance, controlled by a single or a few genes of major effects, to being quantitative resistance, where many genes of small effect influence the resistance (Poland et al., 2009; Poland and Rutkoski, 2016). Marker assisted selection is useful for qualitative resistance (Poland and Rutkoski, 2016) and can be used for introgression or pyramiding of resistance genes, whereas GS is more efficient for improving quantitative resistance. To efficiently use MAS for introgression and/or pyramiding of resistance genes, it is necessary to know the physical location of the genetic variants or quantitative trait loci (QTLs) that affects disease resistance and have access to markers closely linked to the QTL. Genome wide association studies (GWAS) can be used to identify single nucleotide polymorphisms (SNPs) associated with a given trait. Usually, the identified SNPs do not themselves impact the trait but are linked to a causal gene or QTL. GS involves genomic prediction using all available markers simultaneously making it able to include the effects of many QTLs with minor effects (Meuwissen et al., 2001). Knowledge of the location of causal variants or QTLs is not required for GS, but markers covering the full genome are needed to ensure accurate prediction of genomic breeding values.

Employing whole genome sequencing is often neither practical nor essential for GS or MAS. Instead, chip array genotyping, such as a 20,000 SNP array, can be utilized, where the marker density plays a role in the extent of genetic variation that can be captured. (Su et al., 2012; Nielsen et al., 2016). To account for the additive genetic variance not captured by the additive genomic relationship matrix, a residual polygenetic effect can be included in the model. Accounting for the residual additive genetic effect not captured by the markers has been shown to decrease bias of genomic enhanced breeding values and avoid overestimation of the variance of direct genetic values in dairy cattle (Liu et al., 2011). In analysis of data where epistatic and/or dominance effects also impact the phenotype, but are not modelled, these may also be captured by the residual polygenetic effect. Epistasis and dominance lead to heterosis which is expressed in all hybrids. To utilize the existence of epistasis and dominance in rye breeding, the use of hybrid varieties derived from crosses of inbred lines, typically from different heterotic groups, is common.

Modern commercial varieties of rye that are based on the Gülzow type of male-sterility often consist of 3-way hybrids from a cross between lines from a restorer population carrying male-fertility restoring genes (*Rf*) and a 2-way hybrid. The 2-way hybrids stem from a cross between lines from a cytoplasmic male-sterile (CMS) population and a non-restorer germplasm (NRG) population from the same germplasm (i.e., heterotic group) as the CMS. The CMS/NRG heterotic group differs from the heterotic group of the restorer lines (Wilde and Miedaner, 2021). Genetic improvements for scald resistance and other relevant traits are therefore aiming to improve the performance of hybrids rather than the performance of the component lines. Consequently, phenotypes are relatively abundant for the commercially relevant hybrids and collected more sparingly for the parental component lines. However, genotyping is done on the component lines for multiple reasons. Firstly, genetic changes are made in the component lines. Genes of interest in hybrids must therefore be related back to the component lines. Secondly, genotyping of components lines allows for predicting the performance of hybrids from crosses that have not been evaluated yet. Thirdly, if component lines are fully inbred there is no gain in information by genotyping the hybrids as the hybrids’ genotypes would be 100% predictable based on the parental genotypes. However, even if parental lines are not fully inbred there is limited gain in genotyping the offspring as each crossing produces many seeds and they will have all the alleles present in the parental lines in the frequencies they are introduced with (disregarding drift and natural selection). Thus, based on the genotypes of the component lines, the expected heterozygosity can be determined, and the average genotype of the hybrids can be inferred.

For GS, the use of phenotypes from hybrids and genotypes from components lines can be done by including either a combined genomic relationship matrix for all component lines, a matrix for each heterotic group or a matrix for each component line. Which one to use depends on whether the CMS, NGR and restorers have the same genetic variance, or the genetic variances differ between the heterotic group with CMS and NGR and the heterotic group of the restorers or the genetic variances differs between the CMS, NGR and restorers. For MAS, utilization of phenotypes from hybrids and genotypes from component lines in GWAS can be done by inferring the genotype of the hybrid from the genotypes of the components. Using this method, multiple protein-coding genes associated with plant height, heading date, agronomic and grain quality traits have been identified in rye (Siekmann et al., 2021). Disease resistance associated SNPs have been identified for leaf rust (caused by *Puccinia recondita*) and powdery mildew (caused by *Blumeria graminis*) in rye by GWAS on component lines with phenotypes and genotypes from the component lines themselves (Vendelbo et al., 2021), but no reported GWAS studies have used the inferred genotypes of hybrids along with the hybrid phenotypes for identifying SNPs associated with disease resistance in rye. Furthermore, to our knowledge, scald resistance genes have not been reported in either hybrid or component rye. Moreover, the feasibility of GS or MAS has not been evaluated specifically for scald resistance in rye. Thus, there is a need to investigate the genetic architecture of scald resistance in rye and to assess the appropriate method for improving genetic scald resistance in rye.

The aims of the study were to develop models for prediction of genomic values for scald resistance, to identify markers associated with scald resistance in 3-way hybrids of an elite rye breeding program, and to elucidate the genetic architecture of scald resistance in hybrid rye. The outcome of this study will be useful to develop more resilient and sustainable rye cultivars that can meet the growing demand for food and other rye products while reducing the loss from diseases and the dependency on fungicides which feeds into the EU Farm to Fork strategy.

## 2 Materials and methods

### 2.1 Field trials

Three-way hybrids and advanced generations of parental components from the winter rye breeding program of the Nordic Seed company were tested and phenotyped for resistance to scald (*R. Secalis*) in field trials at Aarhus University, Flakkebjerg (Zealand, Denmark) and at Nordic Seed’s facilities in Nienstädt (Germany) and Dyngby (Jutland, Denmark) in two growing seasons, 2020/2021 and 2021/2022. Resistant control varieties included Stannos, KWS Tayo and R3966, and susceptible control varieties included KWS Vinetto and DH386.

#### 2.1.1 Aarhus University Flakkebjerg

At Aarhus University Flakkebjerg, the field trials were sown in a seedmatic design consisting of 1 m^2^ plots (20 cm spacing between plots) of 6 individual 1 m rows with complete randomization of rye lines in two replicates organized in two adjacent blocks. In 2020/2021, a total of 323 entries were sown out on September 23^rd^, 2020, and evaluated in a field site in Flakkebjerg (pre-crop: winter oilseed rape). Each block (replicate) consisted of 6 sowing lids (2 lids by 3 lids, approximately 2 m distance between lids) with 15 plots each. Each plot contained four different rye lines in row 1, 3, 4 and 6 (rows 2 and 5 were spreader rows for later leaf rust inoculation), which were exposed to natural infection by *R. secalis* during winter and spring. Favorable weather conditions with moist and high humidity during spring and early summer 2021 resulted in fast disease development and up to high levels of scald infection. Scald was assessed on June 3^rd^, 2021, at growth stage approx. 57-59 on the BBCH decimal scale (Zadoks et al., 1974; Lancashire et al., 1991).

In 2021/2022, a total of 349 lines were sown out on September 28^th^, 2021, and tested in a similar field trial layout applied on a different field location in Flakkebjerg (pre-crop: grass for seed), but without leaf rust spreader rows, thus, each plot contained 6 individual rows with rye lines. A new complete randomization of lines was applied. Due to each plot having 6 rye lines for testing, the number of lids in each block was reduced from 6 in 2021 to 4 in 2022 (2 lids by 2 lids, approximately 2 m distance between lids) with 15 plots each. Artificial inoculation of *R. secalis* was conducted in this trial using straw (rye variety KWS Binnto) with scald, caused by natural infection by the fungus in a previous year. The straw was then cut and stored until use where it was evenly distributed on the trial area on December 6th, 2021. Medium to high levels of scald were observed in May-June 2022, allowing two disease assessments on June 13^th^ and June 20^th^ at growth stages 60-69 and 70-79, respectively.

In both field trial years at Flakkebjerg local practices were followed without fungicide application. Each line was assessed for scald susceptibility by recording the level of scald infection on leaves (scald lesions cover in percentage) using a standardized assessment scale, which has been applied for characterizing disease susceptibility in cereals by the Danish agricultural advisory services (scores 0-100%) which is comparable to the assessment scale often used by Danish plant breeders (linear scores 1-9) (Pinnschmidt et al., 2006; SEGES Innovation, 2023). See **Supplementary Table S1** for the conversion table.

#### 2.1.2 Nordic Seed Nienstädt and Dyngby

At Nordic Seed Nienstädt and Dyngby locations, the field trial layout was seedmatic trial with three replicates per genotype following a randomized block design with replicates organized in separate blocks. The sowing was done by a 6-row single row seeder in plots (width: 150 cm; length: 100 cm; row spacing: 30 cm; plot spacing 50cm). Per plot three genotypes were sown resulting in two rows per genotype. Sowing was done in the 3^rd^ week of September in Dyngby, and 1^st^ week of October in Nienstädt.

The seedmatic field trial followed local practices without fungicide application. Scald infection occurred naturally and were scored using a 1-9 scale (1 being low disease prevalence) in accordance with the guidelines provided by the German Federal Plant Variety Office (Bundessortenamt, 2016).

### 2.2 DNA extraction and genotyping

Genotyping for the current study was done as part of the standard genotyping scheme at Nordic Seed. Genotyping was performed on the component lines and not on hybrids. The plant material for DNA extraction was germinated in greenhouse facilities of Nordic Seed A/S under controlled temperature and light conditions. The seedlings were cultivated under 16 h of daylight at 18–24°C and 8 h of darkness at 14–16°C. After seven days, the lowest sections of two coleoptiles and primary leaves were excised and stored in a 96-well Micro-Dilution Tube System (STARLAB International GmbH) with glass beads. The plant tissue samples were stored at −20°C for two days before freeze-drying for an additional two days. The DNA was extracted using an adapted SDS-based method as described by (Pallotta et al., 2003). The quality of the DNA was evaluated by measuring its concentration and 260/280 nm absorption ratio using an Epoch TM microplate spectrophotometer (Biotek^®^ Instruments, Winooski, V.T., USA). Fragmentation of the genomic DNA was assessed by size separation on a 1.2% (w/v) agarose gel.

Genotyping was performed on good quality DNA from each genotype included in this study using a custom Illumina Infinium 25K wheat and 5K rye SNP array as described by Vendelbo et al. (2020). The mapping position of SNP markers was identified by mapping the marker sequences to the ‘Lo7’ reference genome (Rabanus-Wallace et al., 2021) using the NCBI blastn (v. 2.9.0+, ML, USA) function at a significance threshold of the e-value at 10^−10^ (https://www.ncbi.nlm.nih.gov) and retained only top hits. Markers were filtered for marker allele frequency (MAF) ≥0.05, missing individual score ≤ 0.2, and missing marker score ≤ 0.1 to identify informative markers across all genotyped individuals resulting in a dataset containing 10875 SNP markers and 1811 genotyped individuals.

The datasets used in the current study contained hybrid phenotypes of scald resistance associated with 185 parental genotypes. Due to the genotypes only representing a subset of the total genotyped population some markers were monomorphic for the lines represented in the experimental data. Markers were removed if they were monomorphic in both heterotic groups (CMS/NRG and restorers) as these markers would be monomorphic in the hybrids as well. Markers that were only monomorphic in one heterotic group were kept. Across the two heterotic groups 4789 markers were polymorphic. Within the restorer heterotic group 4749 markers were polymorphic. 2350 markers were polymorphic in the CMS/NRG heterotic group. Missing markers were imputed using the average allele frequency. The genotypes can be found in **Supplementary File S1, Sheets 1-2**.

### 2.3 Data preparation

To be included in the final datasets, the recorded hybrids needed to be the result of crosses between genotyped component lines. However, in Flakkebjerg, Nienstädt, and Dyngby, the restorer component was not genotyped for 5, 3, and 3 of the hybrids, respectively. Additionally, 52, 30 and 43 hybrids were produced using an ungenotyped NRG component. Consequently, records from these hybrids were excluded, leading to the removal of 66, 99 and 89 records from Flakkebjerg, Nienstädt and Dyngby, respectively. In the dataset from Flakkebjerg, where two scorings were recorded for each line in 2022, only the final measurement was kept for analysis as the disease progression was higher and the genetic potential for scald resistance expressed to a higher degree than in the earlier measurements. The final datasets from each location (**Supplementary File S1 Sheets 3-5**) all contained records from the same 251 hybrids produced by crosses from 166 restorers and 19 CMS/NRG lines. See **Table 1** for summary statistics of the 3 datasets.

**Table 1:** Location, year, number of individuals, number of records, phenotypic mean (SD) and range of scald infection scores in the hybrids.

Location	Year	Hybrids	Records	Mean (SD)	Range
**Flakkebjerg**	2021	118	244	8.67% (7.02)	0.5-37.5% (3-8)
	2022	157	314	8.73% (3.10)	3.0-25.0% (5-7)
**Nienstädt**	2021	118	375	4.78 (0.78)	3-7 (0.5-25%)
	2022	157	483	4.10 (0.60)	3-7 (0.5-25%)
**Dyngby**	2021	118	375	2.23 (1.24)	1-7 (0-25%)
	2022	156	480	1.72 (0.78)	1-5 (0-5%)

Flakkebjerg disease scores are given in percentages (1-9 scale), and Nienstädt and Dyngby scores are given on a 1-9 scale (%).

As the Nienstädt and Dyngby records were obtained from the same trial setup with the same measurement scale, a combined dataset (NS_DB) was also analyzed. The NS_DB dataset contained records from 251 hybrids (**Supplementary File S1 Sheet 6**).

To account for the differences in disease pressure between years the phenotypes were standardized by division by the standard deviation (SD) of the phenotypes within year and location.

To account for micro-level variation in soil structure and local variation in exposure to *R. secalis* infection, spatial effects were also included in all statistical models. In Flakkebjerg, the spatial effect covered half of each block such that sowing lids that were side-by-side were grouped together (3 lids of 15 plots each in 2021 and 2 lids of 15 plots each in 2022). In Nienstädt, Dyngby, and the combined NS_DB dataset, a moving window based on the x and y coordinates of the plants in the field were constructed covering a quadratic area (7 plots by 3 plots), with the plot containing the focal hybrid at the center.

### 2.4 Genomic prediction models

The phenotypes of hybrids were analyzed using 4 models of which the most comprehensive, termed the full model, was:


$$\text{y}=\text{Xb}+{\text{T}}_{\text{a}\left(1\right)}{\text{g}}_{\text{a}\left(1\right)}+{\text{T}}_{\text{a}\left(2\right)}{\text{g}}_{\text{a}\left(2\right)}+{\text{T}}_{\text{aa}\left(1\right)}{\text{g}}_{\text{aa}\left(1\right)}+{\text{T}}_{\text{aa}\left(2\right)}{\text{g}}_{\text{aa}\left(2\right)}+{\text{T}}_{\text{aa}\left(3\right)}{\text{g}}_{\text{aa}\left(3\right)}+{\text{T}}_{\text{D}\left(2\right)}{\text{g}}_{\text{D}\left(2\right)}+{\text{T}}_{\text{D}\left(3\right)}{\text{g}}_{\text{D}\left(3\right)}+{\text{T}}_{\text{l}}\text{l}+{\text{T}}_{\text{c}}\text{c}+\text{e}$$

where y was a vector containing the phenotypes, b was a vector of the fixed effects of year for FL, NS and DB and year-location for NS_DB. ${\text{g}}_{\text{a}\left(1\right)}$ and ${\text{g}}_{\text{a}\left(2\right)}$ were vectors of the random additive genomic effects of parental components from heterotic group 1 (restorer lines) and 2 (CMS and NRG lines), respectively. ${\text{g}}_{\text{aa}\left(1\right)}$ and ${\text{g}}_{\text{aa}\left(2\right)}$ were vectors of the random additive-by-additive epistatic effects of parental components within heterotic group 1 and 2, respectively, and ${\text{g}}_{\text{aa}\left(3\right)}$ was a vector of the random additive-by-additive epistatic effects across heterotic groups. ${\text{g}}_{\text{D}\left(2\right)}$ and ${\text{g}}_{\text{D}\left(3\right)}$ were vectors of the random dominance effects of the maternal cross and the 3-way hybrid, respectively. l and c were vectors of the random line (residual polygenetic effect of each hybrid) and spatial effects (location within plot), and e was a vector of the random residuals. X was the incidence matrix relating phenotypes to fixed effects and ${\text{T}}_{j}$ was the incidence matrix relating phenotypes to random effect *j* ( j∈{a(1), a(2), aa(1), aa(2), aa(3), D(2), D(3), l, c} .

The distribution assumption for the random additive genomic effects of heterotic group 1 (2) was ${\text{g}}_{\text{a}\left(1\right)}∼\text{N}\left(0,{\text{G}}_{\text{a}\left(1\right)}{\text{σ}}_{\text{a}\left(1\right)}^{2}\right)$ [ ${\text{g}}_{\text{A}\left(2\right)}∼\text{N}(0,{\text{G}}_{\text{a}\left(2\right)}\sigma_{\text{a}(2)}^{2})$ ], where ${\text{G}}_{\text{a}\left(1\right)}$ ( ${\text{G}}_{\text{a}\left(2\right)}$ ) was the additive genomic relationship matrix and ${\text{σ}}_{\text{A}\left(1\right)}^{2}$ ( ${\text{σ}}_{\text{A}\left(2\right)}^{2}$ ) was the additive genomic variance of heterotic group 1 (2). The distribution assumption for the random additive-by-additive epistatic effects of heterotic group 1 (2) was ${\text{g}}_{\text{aa}\left(1\right)}∼\text{N}\left(0,{\text{G}}_{\text{aa}\left(1\right)}{\text{σ}}_{\text{aa}\left(1\right)}^{2}\right)$ ( ${\text{g}}_{\text{aa}\left(2\right)}∼\text{N}\left(0,{\text{G}}_{\text{aa}\left(2\right)}{\text{σ}}_{\text{aa}\left(2\right)}^{2}\right)$ ), where ${\text{G}}_{\text{aa}\left(1\right)}$ ( ${\text{G}}_{\text{aa}\left(2\right)}$ ) was the additive-by-additive epistatic genomic relationship matrix and ${\text{σ}}_{\text{aa}\left(1\right)}^{2}$ ( ${\text{σ}}_{\text{aa}\left(2\right)}^{2}$ ) was the additive-by-additive epistatic variance of heterotic group 1 (2). The distribution assumption for the random across heterotic groups additive-by-additive epistatic effects was ${\text{g}}_{\text{aa}\left(3\right)}∼\text{N}\left(0,{\text{G}}_{\text{aa}\left(3\right)}{\text{σ}}_{\text{aa}\left(3\right)}^{2}\right)$ , where ${\text{G}}_{\text{aa}\left(3\right)}$ was the across heterotic groups additive-by-additive epistatic genomic relationship matrix and ${\text{σ}}_{\text{aa}\left(3\right)}^{2}$ was the across heterotic groups additive-by-additive epistatic variance. The distribution assumption for the random dominance effects of the maternal cross (3-way hybrid) was ${\text{g}}_{\text{D}\left(2\right)}∼\text{N}\left(0,{\text{G}}_{\text{D}\left(2\right)}{\text{σ}}_{\text{D}\left(2\right)}^{2}\right)$ ( ${\text{g}}_{\text{D}\left(3\right)}∼\text{N}\left(0,{\text{G}}_{\text{D}\left(3\right)}{\text{σ}}_{\text{D}\left(3\right)}^{2}\right)$ ), where ${\text{G}}_{\text{D}\left(2\right)}$ ( ${\text{G}}_{\text{D}\left(3\right)}$ ) was the dominance genomic relationship matrix and ${\text{σ}}_{\text{D}\left(2\right)}^{2}$ ( ${\text{σ}}_{\text{D}\left(3\right)}^{2}$ ) was the dominance variance of the maternal cross (3-way hybrid). The distribution assumptions for the line and spatial effects were $\text{l}∼\text{N}\left(0,{\text{I}}_{\text{l}}\sigma_{\text{l}}^{2}\right)$ and $\text{c}∼\text{N}\left(0,{\text{I}}_{\text{c}}\sigma_{\text{c}}^{2}\right)$ , where ${\text{I}}_{\text{l}}$ and ${\text{I}}_{\text{c}}$ were identity matrices and $\sigma_{\text{l}}^{2}$ , and $\sigma_{\text{c}}^{2}$ were the line and spatial variances. The distribution assumption for the residuals were $\text{e}∼\text{N}\left(0,\left[\begin{array}{ccc} {\text{I}}_{\text{e}1}{\text{σ}}_{\text{e}1}^{2} & \cdots & 0\\ \vdots & \ddots & \vdots \\ 0 & \cdots & {\text{I}}_{\text{en}}{\text{σ}}_{\text{e}\text{n}}^{2} \end{array}\right]\right)$ , where ${\text{I}}_{\text{ek}}$ was the identity matrix and ${\text{σ}}_{\text{ek}}^{2}$ was the residual variance for year k in FL, DB and NS and year-location k in NS_DB.

The additive genetic, additive-by-additive epistatic and dominance genomic relationship matrices were constructed based on genotypes of the parental lines as described by Kristensen et al. (2023), which enables partitioning of the genomic variance into additive and epistatic variances within each heterotic group as well as epistatic and dominance variances between the heterotic groups.

The remaining models were variations of this model where one or more of the non-additive genomic effects were removed. One model, termed the additive model, included only the additive genomic effects and non-genetic effects shown in the full model, but no non-additive genomic effects. Another model, the epistatic model, included the additive genomic and additive-by-additive epistatic effects as well as the non-genomic effects shown in the full model. A third model, the dominance model, included the additive genomic, dominance and non-genomic effects shown in the full model.

All models were analyzed using DMU v5.6 (Madsen and Jensen, 2013).

Broad and narrow sense heritabilities on the plot and entry-mean (i.e., hybrid-mean) level were calculated for each model and each dataset. Entry-mean heritabilities take the average number of replicated lines into consideration, while the plot level heritability does not but rather calculate a heritability across all records.

On the plot level, narrow sense heritability was calculated as the total additive genomic variance ( ${\hat{\text{σ}}}_{\text{a}}^{2}={\hat{\text{σ}}}_{\text{a}\left(1\right)}^{2}+{\hat{\text{σ}}}_{\text{a}\left(2\right)}^{2}$ ) over the phenotypic variance ( ${\hat{\sigma }}_{\text{P}}^{2}$ ). The phenotypic variance was the sum of all the estimated variances of the random effects, e.g., for the full model: ${\hat{\sigma }}_{\text{P}}^{2}={\hat{\sigma }}_{\text{a}\left(1\right)}^{2}+{\hat{\sigma }}_{\text{a}\left(2\right)}^{2}+{\hat{\sigma }}_{\text{aa}\left(1\right)}^{2}+{\hat{\sigma }}_{\text{aa}\left(2\right)}^{2}+{\hat{\sigma }}_{\text{aa}\left(3\right)}^{2}+{\hat{\sigma }}_{\text{D}\left(2\right)}^{2}+{\hat{\sigma }}_{\text{D}\left(2\right)}^{2}+{\hat{\sigma }}_{\text{l}}^{2}+{\hat{\sigma }}_{c}^{2}*n_{c}+\bar{{\hat{\sigma }}_{\text{e}}^{2}}$ , where $\bar{{\hat{\sigma }}_{\text{e}}^{2}}$ was the average residual variance across year(-location)s ( $\bar{{\hat{\sigma }}_{\text{e}}^{2*}}=\sum_{w=1}^{w=t}\left({\hat{\sigma }}_{{\text{e}}_{w}}^{2}\right)/\;t$ ) and $n_{c}$ was the number of plots included in the moving window used to estimate the spatial effect (twenty one in Nienstädt and Dyngby and one in Flakkebjerg). For the broad sense heritability, the total genetic variance ( ${\hat{\sigma }}_{\text{g}}^{2}={\hat{\sigma }}_{\text{a}\left(1\right)}^{2}+{\hat{\sigma }}_{\text{a}\left(2\right)}^{2}+{\hat{\sigma }}_{\text{aa}\left(1\right)}^{2}+{\hat{\sigma }}_{\text{aa}\left(2\right)}^{2}+{\hat{\sigma }}_{\text{aa}\left(3\right)}^{2}+{\hat{\sigma }}_{\text{D}\left(2\right)}^{2}+{\hat{\sigma }}_{\text{D}\left(2\right)}^{2}+{\hat{\sigma }}_{\text{l}}^{2}$ ) was dived by the phenotypic variance.

To calculate the entry-mean broad and narrow sense heritabilities the phenotypic variance was calculated using $\bar{{\hat{\sigma }}_{\text{e}}^{2*}}=\sum_{w=1}^{w=t}\left({\hat{\sigma }}_{{\text{e}}_{w}}^{2}*r_{w}^{-1}\right)/\;t$ , where *t* is the number of year(-location)s in the dataset and $r_{w}$ was the average number of replicates within year(-location) *w*.

Standard errors of plot and entry-mean heritabilities were calculated using the deltaMethod function of the car package (Fox and Weisberg, 2019) in R (R Core Team, 2020).

#### 2.4.1 Model fit

The fit of the tested models was assessed using Akaike’s information criterion (AIC).


$$AIC_{j}=2k_{j}-2\text{ln}\left(L_{j}\right)$$

where *k* was the number of estimated parameters and *L* was the likelihood of model *j* (full-, additive-, epistatic- or dominance- model). The AIC for all models including non-additive genomic variances was calculated relative to the additive model, such that models with negative AIC had better fit than the additive model.

#### 2.4.2 Cross validation

Leave-hybrid-out (LHO) cross validation was performed for the models used to analyze the hybrid records.

The LHO cross validation schemes was performed within each dataset using the following algorithm:

1. Create a reduced dataset by masking records from a hybrid.
2. Run the full model on the reduced dataset with the variance components previously estimated with the full dataset using the full model.
3. Save the estimated genomic effects for the masked hybrid.
4. Repeat step 2-3 for the additive, epistatic and dominance model.
5. Repeat step 1-5 for the next hybrid.

The accuracy of the models was assessed as the correlation between the corrected phenotypes (see below) and the total genomic effect of the hybrid that was masked in the reduced dataset. The dispersion of predicted genomic values was assessed as the regression coefficient of the corrected phenotypes from the full dataset on the total genetic effects from the reduced dataset.

Corrected phenotypes were calculated by subtracting the fixed effects obtained with the full dataset from the observed phenotypes. The total genetic effect was calculated as the sum of all genomic values in the model estimated with the reduced dataset, e.g., for the full model the estimated genomic value was


$${\hat{\text{g}}}_{ijkl}={\hat{\text{g}}}_{\text{a}\left(1\right),k}+{\hat{\text{g}}}_{\text{a}\left(2\right),\text{k}}+{\hat{\text{g}}}_{\text{a}\left(2\right),m}+{\hat{\text{g}}}_{\text{aa}\left(1\right),k}+{\hat{\text{g}}}_{\text{aa}\left(2\right),l}+{\hat{\text{g}}}_{\text{aa}\left(2\right),\text{m}}+{\hat{\text{g}}}_{\text{aa}\left(3\right),i}+{\hat{\text{g}}}_{\text{D}\left(2\right),lm}+{\hat{\text{g}}}_{\text{D}\left(3\right),i}$$

for hybrid *i*, with restorer *k*, CMS *l* and NRG *m* as parental lines.

### 2.5 Genome wide association study

Genome wide association studies (GWAS) were performed for each dataset. An additive GWAS model was applied by extending the additive model to include a fixed single marker regression on each SNP following:


$$y=Xb+\beta_{i}snp_{i}+T_{A\left(1\right)}g_{A\left(1\right)}+T_{A\left(2\right)}g_{A\left(2\right)}+T_{l}l+T_{c}c+e$$

where $snp_{i}$ was a vector of genotypes for SNP *i* ( i=1…n ), $\beta_{i}$ was the regression coefficient of $snp_{i}$ and the remaining terms were as for the additive model for genomic prediction.

The SNPs used for the single marker regression were on the hybrid level, i.e. they were inferred from the genotypes of each parent using the weighted average, where the restorer genotype was weighted by 0.5 and the CMS and NRG genotypes were both weighted by 0.25. Allele frequencies within the hybrid population were used for the calculation of percentage of phenotypic variance explained (shown below).

To reduce the risk of confounding between the SNP and the additive genomic variance, the additive genomic relationship matrix was reconstructed leaving out the SNPs on the chromosome where the focal SNP is located. The variance components were re-estimated by fitting the additive genomic prediction model, described in the previous section, using the new genomic relationship matrix and the resulting variance components were used as priors for the GWAS analysis. The GWAS analysis was done using DMU v5.6 (Madsen and Jensen, 2013).

#### 2.5.1 Significance and variance explained

In the post-GWAS analysis, quantile-quantile plots and Manhattan plots were constructed. Significance of marker associations were assessed using p-values based on Wald test where the Wald score ( z ) was calculated as


$$W_{i}=\frac{{\hat{\beta }}_{i}}{SE\left({\hat{\beta }}_{i}\right)}$$

The p-values were adjusted for multiple testing using the Benjamini-Hochberg false discovery rate correction (Benjamini and Hochberg, 1995). Marker associations were deemed significant if the adjusted p-value was ≤0.05.

The percentage of phenotypic variance explained (R^2^) by the estimated effect of each significant marker was calculated following:


$$R^{2}=\frac{{\hat{\beta }}_{i}^{2}*2*p\left(1-p\right)}{{\hat{\sigma }}_{P}^{2}}*100\%$$

where ${\hat{\sigma }}_{P}^{2}={\hat{\sigma }}_{a\left(1\right)}^{2}+{\hat{\sigma }}_{a\left(2\right)}^{2}+{\hat{\sigma }}_{l}^{2}+{\hat{\sigma }}_{c}^{2}+\bar{{\hat{\sigma }}_{e}^{2}}$ . $\bar{{\hat{\sigma }}_{e}^{2}}$ was the average residual variance across year(-location)s within each dataset. 95% confidence intervals were constructed for the percentage of phenotypic variance explained by the SNPs based on the upper and lower boundaries of the 95% confidence intervals of $\beta_{i}$ based on the SE of ${\hat{\beta }}_{i}$ .


$$R_{CI}^{2}=\frac{\left({\hat{\beta }}_{i}^{2}\pm 1.96*SE\left({\hat{\beta }}_{i}\right)\right)*2*p\left(1-p\right)}{\sigma_{P}^{2}}*100\%$$

Significant markers were defined as associated with a major QTL if their R^2^≥10% in more than one location.

## 3 Results

Overall, the levels of scald infection varied more between locations than between years. The heritabilities (based on the total additive genetic variance) ranged from 0.26-0.76 for the additive model, suggesting that improving the level of resistance via hybrid breeding programs is possible through genomic selection. The hybrid rye revealed a high level of genomic variability for resistance to scald in the restorer heterotic group. The LHO cross validation and AIC model fit criteria both identified the additive model as the best fitting genomic model for the tested population. The GWAS identified eleven markers associated with QTLs of minor effects and one marker associated with a QTL of major effect, showing that scald resistance is a quantitative trait influenced by many minor QTLs and one or a few major QTL.

### 3.1 Phenotypic characterization of scald

**Figure 1:**
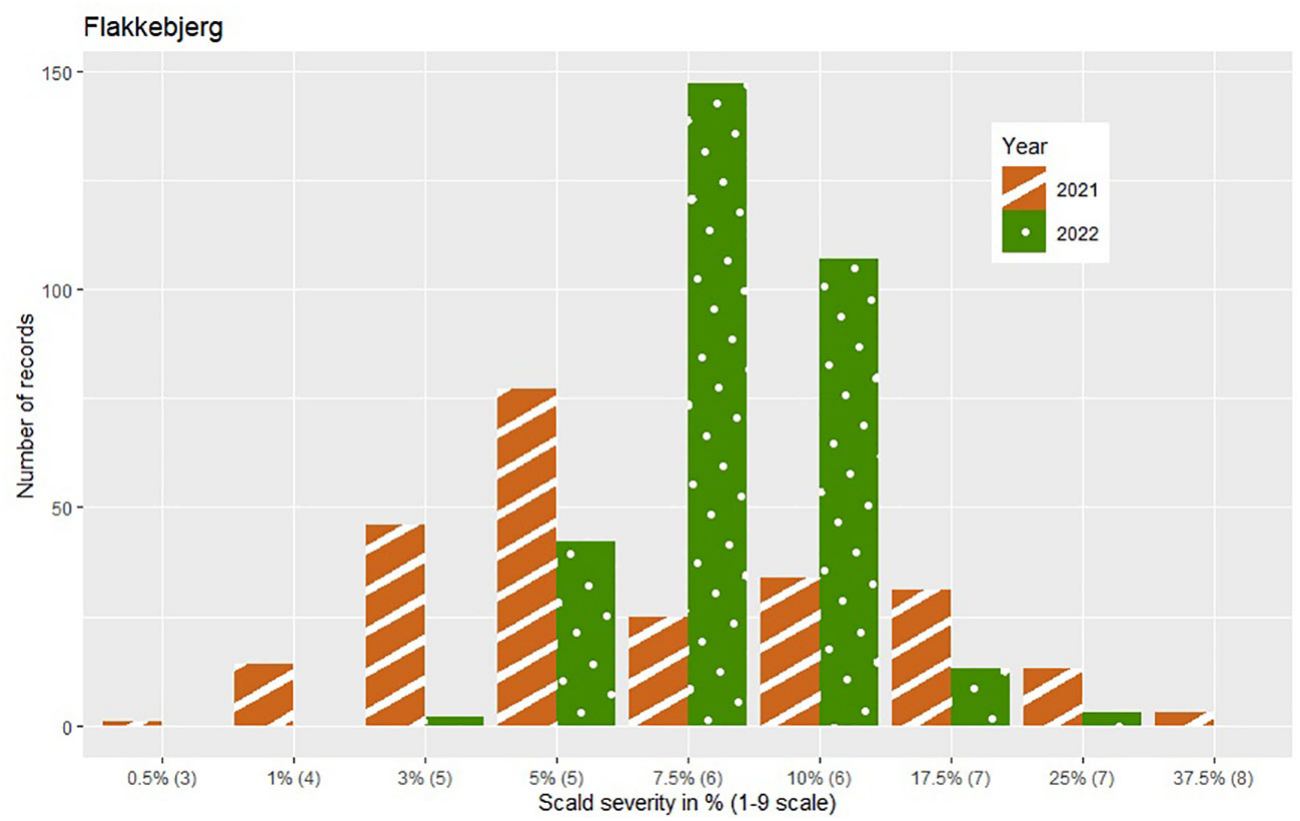
Number of hybrids for each severity level (ranging from 1-37.5%) within year for the hybrids tested in Flakkebjerg. Only hybrids that were included in the analysis are presented. The category of the 1-9 scale corresponding to the severity level in % is given in brackets on the x-axis.

**Figure 2:**
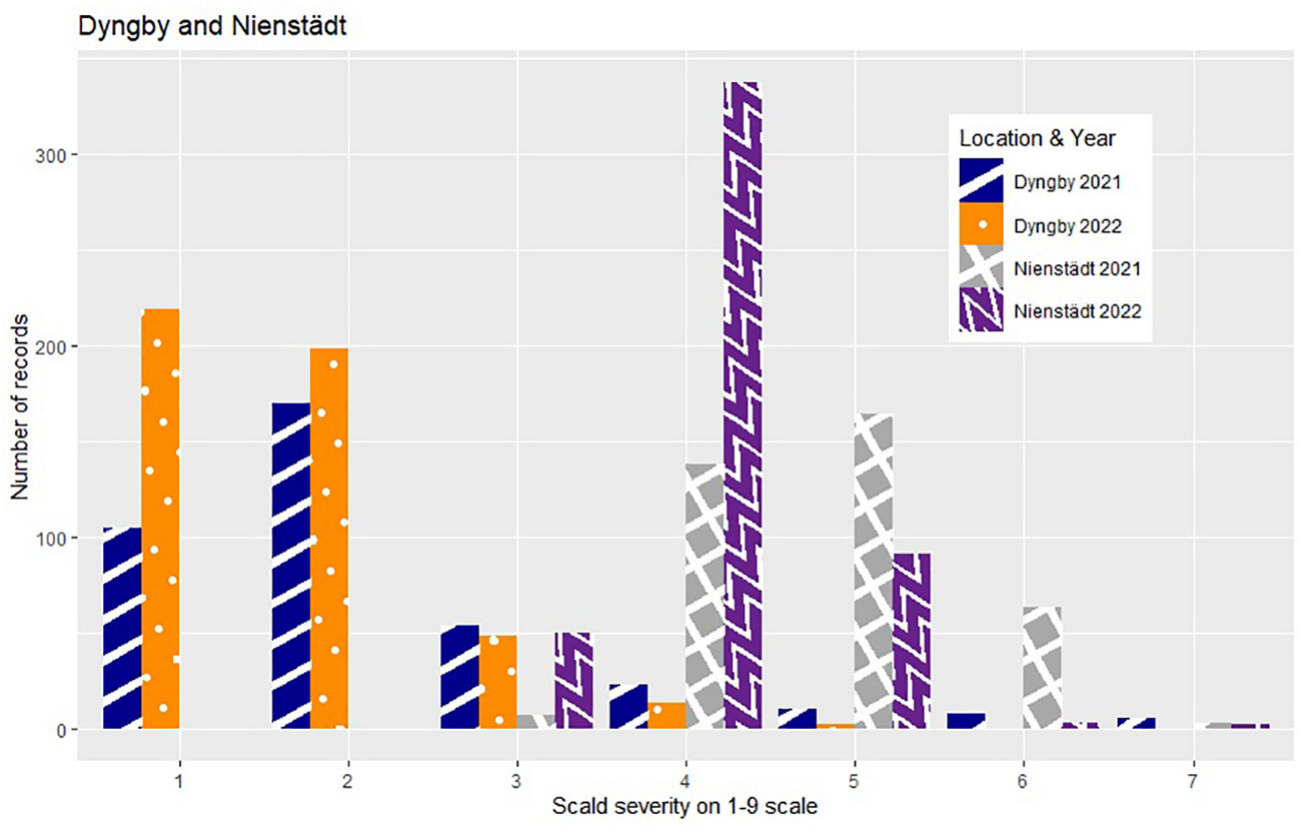
Number of hybrids for each severity level (ranging from 1-9) within year for the hybrids tested in Nienstädt and Dyngby. Only hybrids that were included in the analysis are presented.

The phenotypic expression of scald susceptibility varied between locations with generally lower mean levels of scald infection in Dyngby (**Table 1**) than Nienstädt (**Table 1**) and Flakkebjerg (**Table 1** in %, mean on the 1-9 scale = 5.62 in 2021 and 5.93 in 2022). The mean scald infection levels across years were similar within location. The distribution was approximately normal in both years in Flakkebjerg and Nienstädt (**Figures 1**, **2**). In Dyngby, only the right tail of the normal distribution was expressed in both years, due to the lower disease levels.

**Figure 3:**
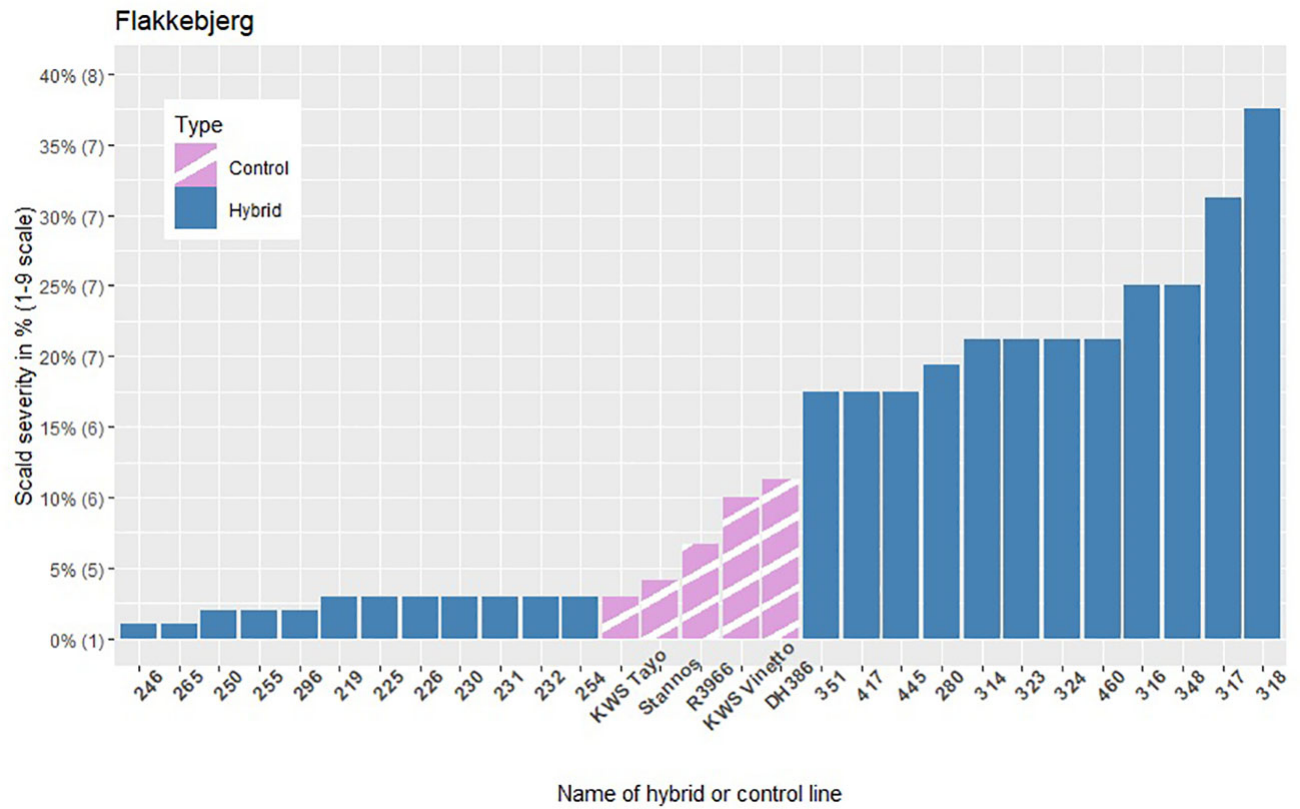
The scald infection severity of the 5% least and 5% most infected hybrids compared to the controls in Flakkebjerg. Only hybrids that were included in the analysis are presented. The category of the 1-9 scale corresponding to the severity level in % is given in brackets on the y-axis.

**Figure 4:**
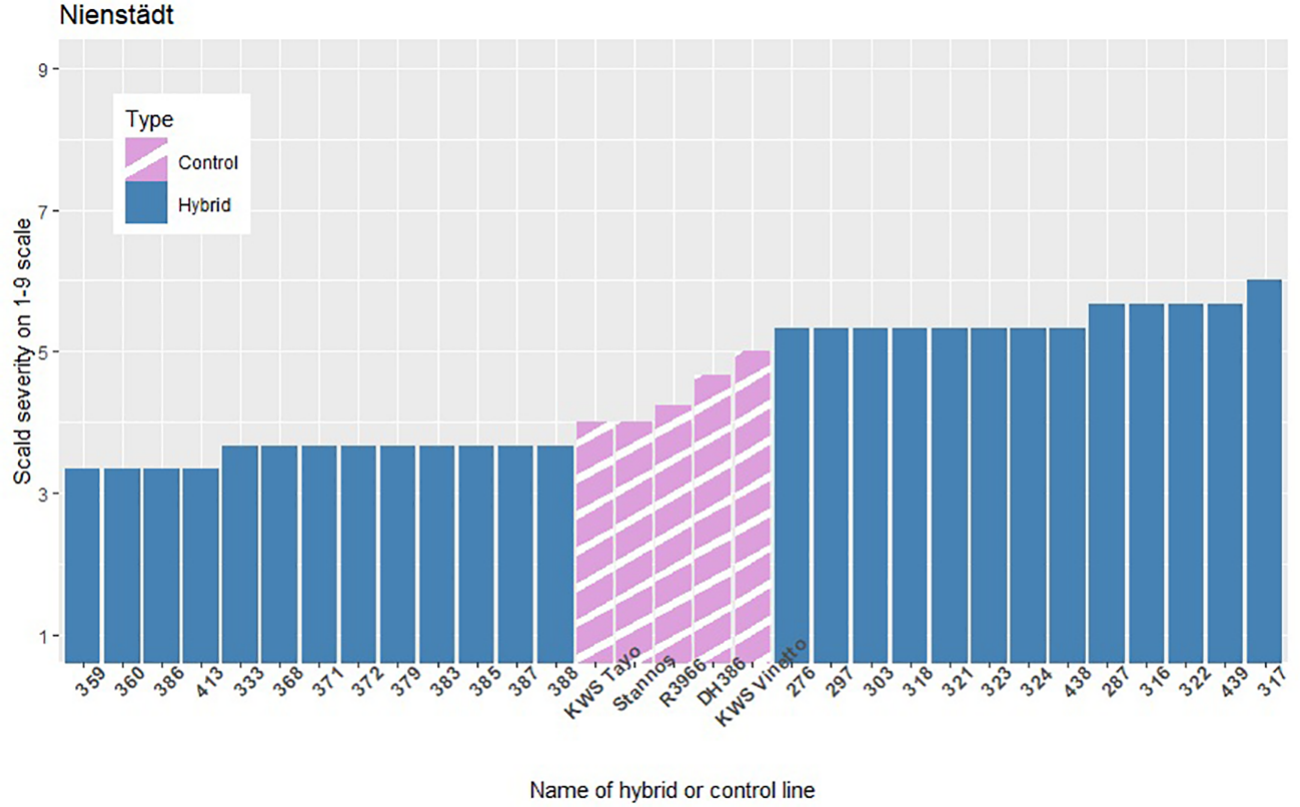
The scald infection severity of the 5% least and 5% most infected hybrids compared to the controls in Nienstädt. Only hybrids that were included in the analysis are presented.

**Figure 5:**
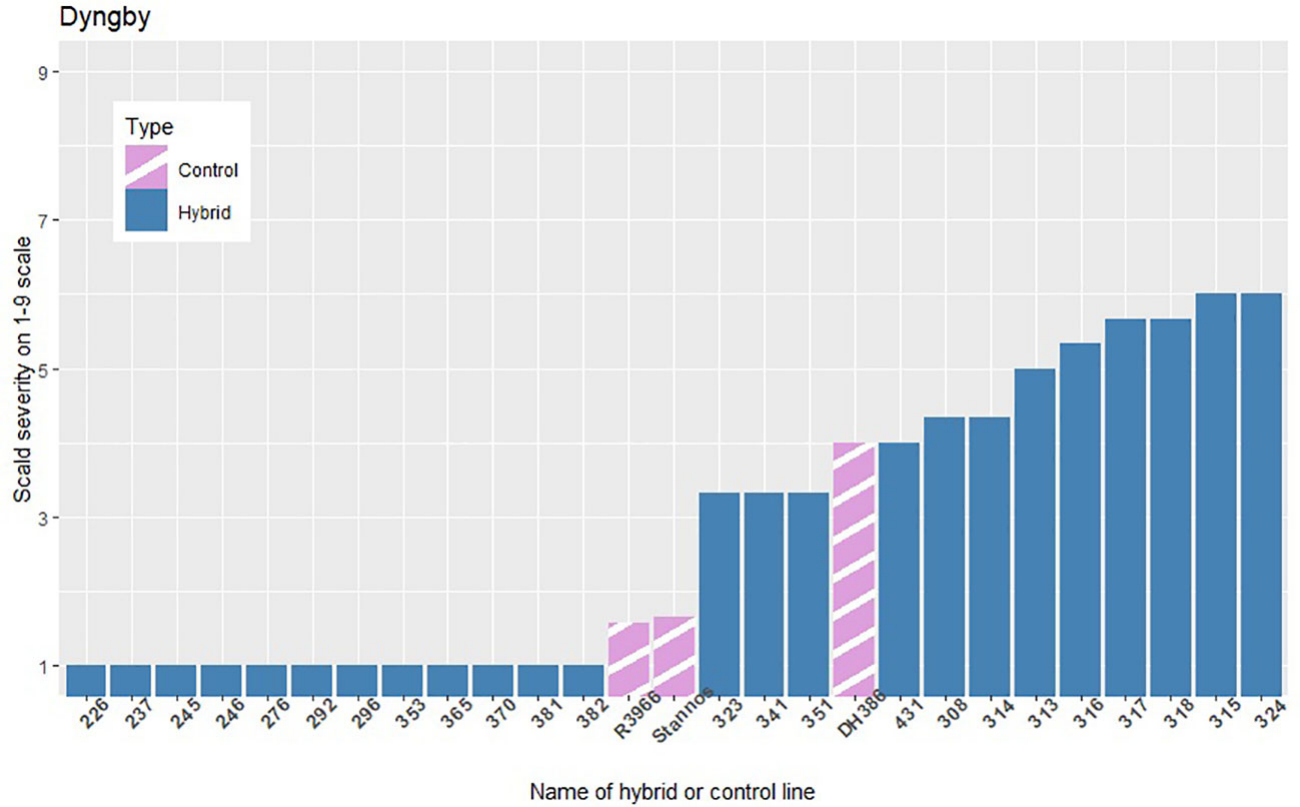
The scald infection severity of the 5% least and 5% most infected hybrids compared to the controls in Dyngby. Only hybrids that were included in the analysis are presented.

The infection levels of the 5% least and 5% most infected hybrids based on the average of the replicates within each location are shown in **Figures 3**–**5**, along with the average infection levels of the control lines used in each location. In Flakkebjerg and Nienstädt the 5% least and 5% most infected hybrids have infection levels below and above the infection levels of the control lines, respectively. In Dyngby, the susceptible control DH386 had higher infection level than the hybrids 343, 341 and 351.

In Nienstädt and Flakkebjerg the infection levels of the 5% least infected hybrids were higher than those in Dyngby due to lower scald levels in Dyngby. There was no overlap between the 5% least infected hybrids in Nienstädt and the other two locations. Meanwhile, 3 of the 5% least infected hybrids in Dyngby were also among the 5% least infected in Flakkebjerg, specifically hybrids 226, 246 and 296. Hybrids 316, 317, 318, 323 and 324 were among the 5% most infected hybrids in all locations. Hybrids 314 and 351 were also among the 5% most infected hybrids in both Flakkebjerg and Dyngby, but not Nienstädt.

### 3.2 Genomic models

As the raw phenotypes were standardized to a variance of 1, variance estimates were approximately variance proportions and therefore generally small. SEs of variance components were generally high relative to the estimates, and generally increased with increasing complexity of models used.

#### 3.2.1 Variances and heritabilities

The broad sense heritabilities were moderate to high on the plot level and high on the entry-mean level in all models in all datasets (**Table 2**). The broad sense heritability varied minimally (± 0.03) between models within dataset. The narrow sense heritability was moderate in the additive and dominance models for all datasets, but low and not significantly different from zero in the epistatic and full models for all datasets.

**Table 2:** Estimated broad and narrow sense heritabilities on the plot and entry-mean level for the hybrid records from Flakkebjerg, Nienstädt and Dyngby, and the combined Nienstädt and Dyngby dataset (NS_DB) using different models.

Dataset	Level	Heritability type	Additive	Epistatic	Dominance	Full
**Flakkebjerg**	Plot	Broad	0.65 (0.03)	0.63 (0.03)	0.65 (0.03)	0.64 (0.03)
		Narrow	0.26 (0.06)	0.03 (0.08)	0.26 (0.07)	0.05 (0.09)
	Entry	Broad	0.76 (0.02)	0.74 (0.02)	0.76 (0.02)	0.75 (0.02)
		Narrow	0.30 (0.07)	0.04 (0.09)	0.30 (0.08)	0.06 (0.10)
**Nienstädt**	Plot	Broad	0.38 (0.06)	0.37 (0.05)	0.37 (0.06)	0.36 (0.05)
		Narrow	0.33 (0.06)	0.06 (0.10)	0.32 (0.07)	0.06 (0.09)
	Entry	Broad	0.64 (0.05)	0.63 (0.05)	0.63 (0.06)	0.62 (0.05)
		Narrow	0.55 (0.08)	0.11 (0.16)	0.54 (0.08)	0.11 (0.16)
**Dyngby**	Plot	Broad	0.45 (0.04)	0.42 (0.04)	0.44 (0.04)	0.42 (0.04)
		Narrow	0.30 (0.05)	0.03 (0.07)	0.24 (0.06)	0.03 (0.07)
	Entry	Broad	0.72 (0.03)	0.69 (0.03)	0.71 (0.03)	0.69 (0.03)
		Narrow	0.47 (0.06)	0.05 (0.11)	0.38 (0.08)	0.05 (0.11)
**NS_DB**	Plot	Broad	0.32 (0.05)	0.31 (0.05)	0.32 (0.05)	0.31 (0.05)
		Narrow	0.26 (0.05)	0.05 (0.07)	0.25 (0.06)	0.04 (0.05)
	Entry	Broad	0.59 (0.05)	0.57 (0.05)	0.59 (0.05)	0.57 (0.05)
		Narrow	0.48 (0.07)	0.10 (0.13)	0.46 (0.08)	0.10 (0.13)

The additive genomic variance of the restorer heterotic group had 95% confidence intervals not overlapping zero, and was therefore considered significantly different from zero, in all datasets for the additive and the dominance models, but not the epistatic or full models (**Supplementary Tables S2-5**). The additive genomic variance of the CMS/NRG heterotic group was not significant in any dataset in any model.

The additive-by-additive epistatic variance in the 3-way hybrids was found to be significant in the records from Dyngby in both the epistatic and full models. The remaining non-additive genomic variances were not significant in any dataset for any model.

The line variance was significantly different from zero in all models for the records from Flakkebjerg and Dyngby, but not for any models for the records from Nienstädt and only the additive and dominance models for the combined NS_DB dataset. The line variance generally decreased with the inclusion of non-additive genetic effects in the model, particularly when additive-by-additive epistasis was included.

The spatial variances were significant in all the models and datasets and were consistent across models within dataset. The spatial variance was similar in Flakkebjerg and Dyngby, but higher in the data from Nienstädt relative to the other locations. In the combined Nienstädt and Dyngby dataset, the spatial variance fell in between that estimated in Nienstädt and in Dyngby.

The residual variances were consistent (± 0.01) across models within year or year-location in each dataset. There was heterogeneity of the residual variance across years in all locations, with Dyngby having the largest difference in residual variances across years. This was reflected in the residual variance across year-locations in the combined NS_DB dataset, with Dyngby 2022 having the lowest and Dyngby 2021 having the highest residual variance.

In summary, there were significant additive genomic variances of the restorer heterotic group in the additive and dominance models for all datasets. There were no significant additive genomic variance of the CMS/NRG heterotic group variances in any model for any dataset and the non-additive genomic variances were generally also non-significant.

#### 3.2.2 Model fit and cross validation

The fit of the tested models assessed using Akaike’s information criterion (AIC) showed the additive model to be the best fitting model for the hybrid records from all datasets.

The LHO cross validation for the additive model showed moderate to high accuracies and no significant over- or underdispersion of predicted genomic values (**Supplementary Table S5**). In the datasets from Nienstädt and Dyngby and the combined Nienstädt and Dyngby dataset, the epistatic, dominance or full models have accuracies of ~ 0 and considerable over- or under-dispersion of genomic values in all datasets for all models. The SEs of the regression coefficient were high, and the over- or under-dispersion was therefore not statistically significantly different from 1. In the dataset from Flakkebjerg, all estimated genomic values were 0 in both the epistatic and dominance models, and it was therefore not possible to calculate accuracy or dispersion of genomic values. In the full model the accuracies were ~ 0 and the genomic values were under dispersed, but with large SEs rendering the regression coefficient not significantly different from 1.

In summary, the AIC and cross validation showed that the additive model was the preferred model for analyzing the current dataset.

### 3.3 GWAS

The GWAS was performed on each rye dataset and identified twelve SNPs associated with scald resistance spread across all chromosomes, except chromosome 7R (**Table 3**). The twelve SNPs were present in Flakkebjerg (4 SNPs), Dyngby (10 SNPs) and/or the combined Nienstädt and Dyngby datasets (5 SNPs). No SNPs were significantly associated with scald resistance in the Nienstädt dataset.

**Table 3:** Marker ID, chromosome number (Chr.no), and sequence length in base pairs (bp), allele frequency in the hybrid population, estimated regression coefficient ( $\hat{\beta }$ , SE) and percentage of the phenotypic variance ( $\sigma_{P}^{2}$ ) explained by the marker [95% confidence interval] within Flakkebjerg, Dyngby and the combined Nienstädt and Dyngby dataset (NS_DB).

Marker^+^	Chr.no	Length (bp)	Frequency	$\hat{\beta }$	% of $\;\sigma_{P}^{2}$
Flakkebjerg					
46	1R	102	0.525	1.28 (0.31)	88 [25-339]
*3814*	1R	72	0.011	2.24 (0.47)	12 [4-45]
2251	2R	121	0.004	2.91 (0.68)	7 [2-28]
**2208**	5R	121	0.013	2.05 (0.48)	12 [3-45]
Dyngby					
758	1R	102	0.014	1.20 (0.27)	7 [2-27]
*3814*	1R	72	0.011	1.18 (0.28)	5 [2-21]
*1223*	1R	72	0.008	1.45 (0.31)	6 [2-23]
*3022*	2R	72	0.012	1.70 (0.32)	12 [5-45]
1664	3R	72	0.006	1.60 (0.38)	5 [1-21]
*3331*	4R	171	0.008	1.47 (0.32)	6 [2-23]
1707	5R	72	0.049	0.84 (0.17)	11 [4-44]
**2208**	5R	121	0.013	1.57 (0.30)	11 [4-43]
*1398*	6R	121	0.211	0.97 (0.24)	56 [15-216]
2535	6R	120	0.233	0.70 (0.17)	31 [8-120]
NS_DB					
*1223*	1R	72	0.008	1.05 (0.24)	3 [0.94-11.77]
*3022*	2R	72	0.012	1.03 (0.24)	4 [1.20-15.26]
*3331*	4R	171	0.008	1.05 (0.24)	3 [0.91-11.55]
**2208**	5R	121	0.013	1.00 (0.22)	4 [1.39-17.04]
*1398*	6R	121	0.211	0.89 (0.20)	46 [14.60-176.87]

^+^Markers in italic are present in two datasets, markers in bold are present in three datasets.

Two of the significant SNPs in Flakkebjerg were also significant in Dyngby (SNP 2208 and 3814). All the SNPs detected that were found significant in the combined Nienstädt and Dyngby dataset were also significant in the Dyngby dataset. These all had smaller estimated effects and percentage of phenotypic variance explained in the combined Nienstädt and Dyngby dataset compared to the Dyngby dataset. These SNPs were assumed significant in the combined dataset due to their impact in the Dyngby dataset since no SNPs were significant in the Nienstädt dataset.

The regression coefficients were all positive meaning the focal allele (the minor allele for all but one SNP) was shown to decrease scald resistance. Frequencies were<0.05 for nine of the twelve SNPs showing that the major allele is almost fixed in the rye population. These SNPs each explained 3-12% of the phenotypic variance. The remaining three SNPs had MAF of 0.21-0.53 and each explained a substantial percentage (31-88%) of phenotypic variance.

## 4 Discussion

In this study, the genetic architecture of scald resistance in 3-way hybrid rye was investigated and markers potentially associated with scald resistance genes were identified.

Both the narrow and broad sense heritabilities observed in the additive model were moderate to high (0.26-0.55 for narrow sense and 0.32-0.76 for broad sense). While there is no report on the heritability of scald resistance in rye in the literature, studies of scald in barley have shown broad sense heritabilities ranging from 0.54-0.97 (Spaner et al., 1998; Aoki et al., 2011; Fériani et al., 2012; Xi et al., 2019; Zantinge et al., 2019). While the heritabilities presented in the current study are from rye rather than barley, they are comparable to those reported for barley, indicating that response to selection on scald resistance in hybrid rye should be similar to those of barley assuming the same selection pressure. Laidig et al. (2021) examined the breeding progress of multiple traits in German cereals, including hybrid winter rye and winter barley. Winter barley and hybrid winter rye both showed significant improvement in scald resistance, though both the variety mean resistance and the progress in resistance were superior in the winter barley population (Laidig et al., 2021). This may be due to differences in the time of inclusion of selection on scald resistance in the breeding goals of rye and barley, due to scald resistance having higher relative weighting in the breeding goals in barley than rye or due to the genetic variance of scald resistance in barley being higher. However, it clearly shows that scald resistance in hybrid rye can be improved through selection.

Most of the additive genomic variance detected was attributed to the restorer component lines. The additive genomic variance in the hybrids attributed to the CMS/NRG heterotic group was not significantly different from 0 in any dataset or model. This was likely due to the group only containing 19 closely related lines and having a high proportion of monomorphic markers (51%) compared to the restorer heterotic group (0.8% monomorphic markers). Vendelbo et al. (2020) examined the germplasm of 250 restorer component lines, 119 NRG, and 7 CMS lines which are used by Nordic Seed A/S for hybrid rye breeding using 4419 SNPs. They reported 42% and 0.9% monomorphic markers in the CMS/NRG and restorer heterotic groups, respectively. This shows that even for the wider population of CMS/NRG lines owned by Nordic Seed A/S there is high genetic similarity between lines. Additionally, due to historical reasons approximately half of the hybrids in the current study had the same CMS/NRG line as crossing parent. It is therefore not surprising that only little of the genomic variance expressed in the hybrids was attributed to the CMS/NRG heterotic group.

Although a considerable number of hybrids were tested, CMS/NRG parental lines posed a challenge, as these lines were both few and closely related. Furthermore, a single CMS/NRG line served as the parent for roughly half of the hybrids, further contributing to the closely related nature of the tested hybrids. Consequently, the data suffers from low statistical power due to these factors.

The lack of power in the data has caused issues with orthogonality in the data, reducing the ability of the models to accurately separate the non-additive genomic variances from each other and, especially, separate the additive-by-additive epistatic variances from the additive genomic variances. This causes the additive genomic variance to decrease when additive-by-additive genetic variances were included in the models. Similar issues with lack of orthogonality of additive and additive-by-additive genomic variances have been found for yield in inbred wheat varieties (Raffo et al., 2022). This is also reflected in the reduced fit and prediction ability of the models with non-additive genomic effects compared to the additive model.

The lack of power and resulting orthogonality issues means that while no non-additive genomic variances were consistently found significant across datasets, it cannot be said with certainty that no such variances are impacting scald resistance in hybrid rye. Significant additive-by-additive epistatic and dominance variances have been found for yield, protein content and lodging in internal analysis of field trials involving the Nordic Seed’s hybrid winter rye population (Sarup, pers. comm.), showing that non-additive genomic variances are present in this population. However, previous studies of disease traits in rye have found little impact of non-additive effects (Miedaner and Geiger, 1996; Kolasińska and Węgrzyn, 2003; Sarup, pers. comm.). In future studies it would be beneficial to examine more diverse populations to determine whether non-additive genetic effects impact scald resistance.

The GWAS identified twelve markers that were significantly associated with scald resistance in hybrids. Depending on the definition of major QTL, some of the significant markers could be linked to a major QTL. In this study, markers were considered as associated with major QTLs if they explain 10% or more of the phenotypic variance in more than one location [similar to Pilet-Nayel et al. (2002)]. The reason being that breeding companies generally wish to sell to a wide range of markets and environments and thus, using markers that were only significantly associated with the trait and/or only have major impacts in one location for MAS is not ideal (Fériani et al., 2020). Per this definition, one of the significant markers was considered as associated with a major QTL, while eleven were associated with minor QTLs. This indicates that scald resistance in hybrid rye was influenced by many minor QTLs and one major QTL in the studied breeding material, making it a largely quantitative trait.

The reason for the low number of markers that were found significant in multiple locations could be genotype-by-environment interactions (GEI). While not reported for scald in rye, GEI across locations and/or years have been found for scald in barley (Xi et al., 2019; Fériani et al., 2020). In rye, GEI have been found for other fungal diseases including fusarium head blight (caused by *Fusarium* spp.) (Miedaner et al., 2001; Miedaner et al., 2003) and ergot (caused by *Claviceps purpurea*) (Mirdita et al., 2008). Thus, scald resistance could be exhibiting GEI between the three trial locations included in the study.

The results show evidence of GEI in scald resistance between Nienstädt. The additive genomic correlations were 0.28 between the additive genomic effect in Nienstädt and Flakkebjerg and 0.60 between the additive effects of the restorer heterotic group in Nienstädt and Dyngby when analyzing the records using a bivariate additive model (results not shown). The low to moderate genetic correlations indicate re-ranking of genotypes (i.e., the best genotype in one location is not necessarily the best in another location) would be expected between the Nienstädt and the other two locations due to GEI. This was also observed in the phenotypic scald severity where none of the 5% least infected hybrids in Nienstädt (**Figure 4**) were among the 5% least infected hybrids in Flakkebjerg (**Figure 3**) or Dyngby (**Figure 5**). The presence of GEI can result in different QTLs being detected in different environments or QTLs can have environment specific effects, i.e., there are QTL-by-environment interactions. QTL-by-environment interactions have previously been reported for scald resistance in barley (Von Korff et al., 2005; Fériani et al., 2020). It is therefore possible that no markers were found significant in the dataset from Nienstädt due to environment specific minor effects that were below the detection limit of the dataset as discussed previously.

The presence of GEI and environment dependent QTLs may also be the reason why only half of the significant markers in Dyngby were found when the records were combined with the records from Nienstädt. While the difference in the estimated effects of the shared markers were not significant, the consistent decrease in effect when including the records from Nienstädt could indicate that the GEI between the two locations reduces the ability of the GWAS to detect the effect of the markers in the Dyngby dataset.

The results did not show as clear an indication of GEI between the Flakkebjerg and Dyngby datasets as between the Nienstädt and Flakkebjerg or Dyngby datasets. While only two of the four markers that were found significant in Flakkebjerg were found in Dyngby as well, the genomic correlations were greater than 0.95 for both heterotic groups when analyzing the records from Flakkebjerg and Dyngby using a bivariate additive model (results not shown). This is somewhat contradictory but could be due to differences in disease pressure between the two locations, e.g., in 2022, scald severity was low in the naturally infected trial in Dyngby (1-5 on the 1-9 disease scale, or 0-5%) and high in the inoculated field trial in Flakkebjerg (3-25%, or 5-7 on the 1-9 scale). The fact that Nienstädt vs Flakkebjerg or Dyngby shows more GEI than Flakkebjerg vs Dyngby is not surprising given Nienstädt is the most southern location and Flakkebjerg and Dyngby are located fairly close. In future disease tests of new varieties, it is important to have test sites that cover the different environments for which the varieties are to be marketed.

## 5 Conclusion

In this study we showed that scald resistance in hybrid winter rye is a quantitative trait influenced by many minor QTLs and one or a few major QTLs, which is impacted by GEI. The marker associated with the major QTL was nearly fixed for the favorable allele showing little potential for MAS in breeding for scald resistance in rye. Considerable genomic variation was found with moderate to high heritabilities, in combination with moderate to high predictive accuracies in leave one out cross validation. This showed that breeding with GS would be more efficient than MAS for increasing scald resistance in winter rye.

## Data availability statement

The datasets presented in this study can be found in online repositories. The names of the repository/repositories and accession number(s) can be found in the article/**Supplementary Material**.

## Author contributions

MDM: Formal analysis, Methodology, Writing – original draft, Writing – review & editing. PK: Data curation, Methodology, Writing – review & editing. KM: Data curation, Methodology, Writing – review & editing. TT: Data curation, Methodology, Writing – review & editing. MM: Data curation, Methodology, Writing – review & editing. JO: Writing – review & editing. PS: Data curation, Writing – review & editing. AJ: Writing – review & editing. MH: Data curation, Writing – review & editing. JR-A: Data curation, Writing – review & editing. JJ: Formal analysis, Funding acquisition, Methodology, Supervision, Writing – review & editing.
