# Supplementary material for: Scald resistance in hybrid rye (Secale cereale): genomic prediction and GWAS
Source: Front Plant Sci. 2024 Jan 18;15:1306591. doi: 10.3389/fpls.2024.1306591 (PMC10830712; doi:10.3389/fpls.2024.1306591)
Supplement: Supplementary file 2 [file Table_2.docx]

**Supplementary Table S1 Conversion table between the disease severity in % and on the 1-9 scale.**

| **Disease level** | **Disease severity** | |
| --- | --- | --- |
|  | **%** | **1-9 scale** |
| None or trace | 0 | 1 |
|  | 0.1 | 2 |
| Low | 0.5 | 3 |
|  | 1 | 4 |
| Medium | 5 | 5 |
| High | 10 | 6 |
|  | 25 | 7 |
| Very high | 50 | 8 |
|  | 75 | 9 |

Supplementary Table S2 Estimated variance components from the four genomic prediction model from Flakkebjerg.

| Flakkebjerg |  | Additive | Epistatic | Dominance | Full |
| --- | --- | --- | --- | --- | --- |
| Additive genetic | Restorer | 0.24 (0.10) | 0.03 (0.1) | 0.24 (0.11) | 0.04 (0.11) |
|  | CMS+NRG | 0.00 (0.04) | 0.00 (0.06) | 0.00 (0.05) | 0.00 (0.07) |
| Additive-by-additive epistatic | Restorer | - | 0.22 (0.12) | - | 0.22 (0.12) |
|  | CMS+NRG | - | 0.00 (0.03) | - | 0.00 (0.03) |
|  | Hybrid | - | 0.01 (0.06) | - | 0.00 (0.08) |
| Dominance | CMS+NRG | - | - | 0.01 (0.02) | 0.01 (0.02) |
|  | Hybrid | - | - | 0.00 (0.06) | 0.00 (0.07) |
| Line | Hybrid | 0.36 (0.06) | 0.30 (0.06) | 0.35 (0.06) | 0.30 (0.06) |
| Spatial |  | 0.06 (0.02) | 0.06 (0.02) | 0.06 (0.02) | 0.06 (0.02) |
| Residual | 2021 | 0.32 (0.05) | 0.31 (0.05) | 0.31 (0.05) | 0.31 (0.05) |
|  | 2022 | 0.20 (0.03) | 0.21 (0.03) | 0.21 (0.03) | 0.21 (0.03) |

Supplementary Table S3 Estimated variance components from the four genomic prediction model from Nienstädt.

| Nienstädt |  | Additive | Epistatic | Dominance | Full |
| --- | --- | --- | --- | --- | --- |
| Additive genetic | Restorer | 0.08 (0.04) | 0.04 (0.04) | 0.08 (0.04) | 0.03 (0.04) |
|  | CMS+NRG | 0.11 (0.07) | 0.00 (0.07) | 0.10 (0.07) | 0.00 (0.07) |
| Additive-by-additive epistatic | Restorer | - | 0.07 (0.04) | - | 0.07 (0.04) |
|  | CMS+NRG | - | 0.09 (0.05) | - | 0.09 (0.04) |
|  | Hybrid | - | 0.00 (0.03) | - | 0.00 (0.03) |
| Dominance | CMS+NRG | - | - | 0.00 (0.01) | 0.00 (0.01) |
|  | Hybrid | - | - | 0.00 (0.02) | 0.00 (0.02) |
| Line | Hybrid | 0.03 (0.02) | 0.01 (0.02) | 0.03 (0.02) | 0.01 (0.02) |
| Spatial |  | 0.31 (0.03) | 0.31 (0.03) | 0.31 (0.03) | 0.31 (0.03) |
| Residual | 2021 | 0.32 (0.04) | 0.32 (0.04) | 0.32 (0.04) | 0.32 (0.04) |
|  | 2022 | 0.36 (0.04) | 0.35 (0.03) | 0.36 (0.04) | 0.35 (0.03) |

Supplementary Table S4 Estimated variance components from the four genomic prediction model from Dyngby.

| Dyngby |  | ADD | AA | DOM | FULL |
| --- | --- | --- | --- | --- | --- |
| Additive genetic | Restorer | 0.11 (0.04) | 0.02 (0.04) | 0.07 (0.04) | 0.02 (0.04) |
|  | CMS+NRG | 0.06 (0.05) | 0.00 (0.04) | 0.06 (0.05) | 0.00 (0.04) |
| Additive-by-additive epistatic | Restorer | - | 0.06 (0.04) | - | 0.06 (0.04) |
|  | CMS+NRG | - | 0.06 (0.03) | - | 0.06 (0.03) |
|  | Hybrid | - | 0.03 (0.03) | - | 0.03 (0.03) |
| Dominance | CMS+NRG | - | - | 0.00 (0.01) | 0.00 (0.01) |
|  | Hybrid | - | - | 0.09 (0.03) | 0.00 (0.03) |
| Line | Hybrid | 0.09 (0.02) | 0.06 (0.02) | 0.07 (0.02) | 0.06 (0.02) |
| Spatial |  | 0.06 (0.01) | 0.06 (0.01) | 0.06 (0.01) | 0.06 (0.01) |
| Residual | 2021 | 0.18 (0.02) | 0.18 (0.02) | 0.18 (0.02) | 0.18 (0.02) |
|  | 2022 | 0.43 (0.04) | 0.44 (0.04) | 0.44 (0.04) | 0.44 (0.04) |

Supplementary Table S5 Estimated variance components from the four genomic prediction model from the combined Nienstädt and Dyngby dataset (NS_DB).

| NS_DB |  | ADD | AA | DOM | FULL |
| --- | --- | --- | --- | --- | --- |
| Additive genetic | Restorer | 0.06 (0.02) | 0.03 (0.03) | 0.05 (0.02) | 0.03 (0.03) |
|  | CMS+NRG | 0.10 (0.06) | 0.00 (0.06) | 0.10 (0.07) | 0.00 (0.06) |
| Additive-by-additive epistatic | Restorer | - | 0.02 (0.02) | - | 0.02 (0.02) |
|  | CMS+NRG | - | 0.09 (0.04) | - | 0.09 (0.04) |
|  | Hybrid | - | 0.01 (0.02) | - | 0.01 (0.02) |
| Dominance | CMS+NRG | - | - | 0.00 (0.01) | 0.00 (0.01) |
|  | Hybrid | - | - | 0.02 (0.01) | 0.00 (0.02) |
| Line | Hybrid | 0.04 (0.01) | 0.02 (0.01) | 0.03 (0.01) | 0.02 (0.01) |
| Spatial |  | 0.18 (0.02) | 0.18 (0.02) | 018 (0.02) | 0.18 (0.02) |
| Residual | NS2021 | 0.42 (0.04) | 0.42 (0.04) | 0.42 (0.04) | 0.42 (0.04) |
|  | NS2022 | 0.43 (0.04) | 0.43 (0.04) | 0.43 (0.04) | 0.43 (0.04) |
|  | DB2021 | 0.21 (0.02) | 0.21 (0.02) | 0.21 (0.02) | 0.21 (0.02) |
|  | DB2022 | 0.47 (0.04) | 0.47 (0.04) | 0.47 (0.04) | 0.47 (0.04) |

Supplementary Table S6. Akaike’s information criterion (AIC), as well as accuracy and dispersion of the leave-individual-out cross validation for the genomic prediction models for hybrid records from Flakkebjerg, Nienstädt and Dyngby, and the combined Nienstädt and Dyngby dataset (NS_DB).

| **Parameter** | **Level** | **Dataset** | | **Additive** | **Epistatic** | **Dominance** | **Full** |
| --- | --- | --- | --- | --- | --- | --- | --- |
| ΔAIC | | | Flakkebjerg | 0 | 299.02 | 427.87 | 726.94 |
|  |  |  | Nienstädt | 0 | 296.91 | 428.16 | 725.02 |
|  |  |  | Dyngby | 0 | 291.98 | 425.69 | 720.17 |
|  |  |  | NS_DB | 0 | 295.64 | 427.89 | 723.84 |
| Accuracy | Plot | | Flakkebjerg | 0.59 (0.03) | NA | NA | 0.08 (0.04) |
|  |  | | Nienstädt | 0.56 (0.03) | -0.04 (0.03) | -0.04 (0.03) | -0.04 (0.03) |
|  |  | | Dyngby | 0.50 (0.03) | -0.04 (0.03) | -0.07 (0.03) | -0.04 (0.03) |
|  |  | | NS_DB | 0.49 (0.02) | -0.05 (0.02) | -0.06 (0.02) | -0.05 (0.02) |
|  | Entry | | Flakkebjerg | 0.76 (0.04) | NA | NA | 0.11 (0.06) |
|  |  | | Nienstädt | 0.75 (0.04) | -0.01 (0.06) | 0.00 (0.06) | -0.02 (0.06) |
|  |  | | Dyngby | 0.72 (0.04) | -0.07 (0.06) | -0.10 (0.06) | -0.07 (0.06) |
|  |  | | NS_DB | 0.81 (0.04) | -0.05 (0.06) | -0.05 (0.06) | -0.05 (0.06) |
| Dispersion | Plot | | Flakkebjerg | 1.06 (0.06) | NA | NA | 7.37*10^11^ (4.12*10^11^) |
|  |  | | Nienstädt | 1.01 (0.05) | -2.52 (2.04) | -2.36 (2.26) | -2.62 (2.00) |
|  |  | | Dyngby | 0.93 (0.05) | -1.92 (1.47) | -3.51 (1.63) | -1.92 (1.47) |
|  |  | | NS_DB | 0.94 (0.04) | -2.42 (1.22) | -3.23 (1.40) | -2.42 (4.43) |
|  | Entry | | Flakkebjerg | 1.06 (0.06) | NA | NA | 9.43*10^11^ (5.18*10^11^) |
|  |  | | Nienstädt | 1.02 (0.06) | -0.63 (3.56) | 0.25 (3.63) | -0.87 (3.53) |
|  |  | | Dyngby | 0.95 (0.06) | -2.46 (2.33) | -3.69 (2.38) | -2.46 (2.33) |
|  |  | | NS_DB | 0.95 (0.06) | -1.81 (2.27) | -2.17 (2.51) | -1.81 (2.27) |

ΔAIC=AIC_x_-AIC_Additive_
